# Depletion of G9A attenuates imiquimod-induced psoriatic dermatitis via targeting EDAR-NF-κB signaling in keratinocyte

**DOI:** 10.1038/s41419-023-06134-y

**Published:** 2023-09-22

**Authors:** Zhiqin Fang, Yutong Wang, Bo Huang, Xiang Chen, Rundong Jiang, Mingzhu Yin

**Affiliations:** 1 Department of Dermatology, Hunan Engineering Research Center of Skin Health and Disease, Hunan Key Laboratory of Skin Cancer and Psoriasis, Xiangya Clinical Research Center for Cancer Immunotherapy, Xiangya Hospital, Central South University, Changsha, Hunan China; 2 National Clinical Research Center for Geriatric Disorders, Xiangya Hospital, Central South University, Changsha, Hunan China; 3 National Engineering Research Center of Personalized Diagnostic and Therapeutic Technology, Changsha, Hunan China; 4 Clinical Medicine Eight-Year Program, Xiangya School of Medicine, Central South University, Changsha, Hunan China; 5 Clinical Research Center, Medical Pathology Center, Cancer Early Detection and Treatment Center, Chongqing University Three Gorges Hospital, Chongqing University, Wanzhou, Chongqing China; 6 Translational Medicine Research Center, School of Medicine Chongqing University, Shapingba, Chongqing China

**Keywords:** Autoimmunity, Apoptosis

## Abstract

Psoriasis is a common and recurrent inflammatory skin disease characterized by inflammatory cells infiltration of the dermis and excessive proliferation, reduced apoptosis, and abnormal keratosis of the epidermis. In this study, we found that G9A, an important methyltransferase that mainly mediates the mono-methylation (me1) and di-methylation (me2) of histone 3 lysine 9 (H3K9), is highly expressed in lesions of patients with psoriasis and imiquimod (IMQ)-induced psoriasis-like mouse model. Previous studies have shown that G9A is involved in the pathogenesis of various tumors by regulating apoptosis, proliferation, differentiation, and invasion. However, the role of G9A in skin inflammatory diseases such as psoriasis remains unclear. Our data so far suggest that topical administration of G9A inhibitor BIX01294 as well as keratinocyte-specific deletion of G9A greatly alleviated IMQ-induced psoriatic alterations in mice for the first time. Mechanistically, the loss function of G9A causes the downregulation of Ectodysplasin A receptor (EDAR), consequently inhibiting the activation of NF-κB pathway, resulting in impaired proliferation and increased apoptosis of keratinocytes, therefore ameliorating the psoriatic dermatitis induced by IMQ. In total, we show that inhibition of G9A improves psoriatic-like dermatitis mainly by regulating cell proliferation and apoptosis rather than inflammatory processes, and that this molecule may be considered as a potential therapeutic target for keratinocyte hyperproliferative diseases such as psoriasis.

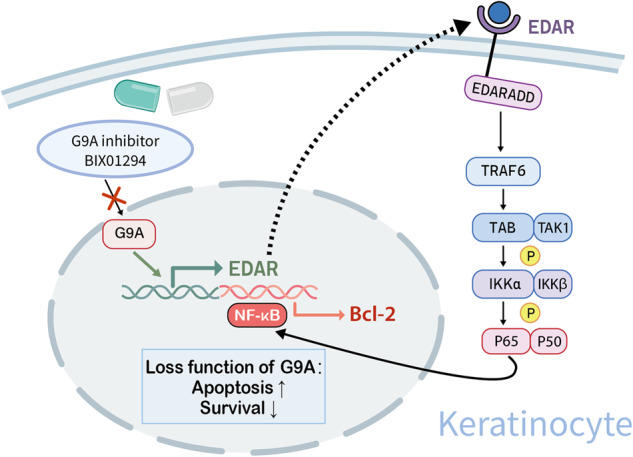

## Introduction

Psoriasis is a prevalent and persistent autoimmune skin disorder, in which skin tissues accompany with abnormal activation of the immune system and over-proliferation of keratinocytes [1–3]. On the other hand, the resistance of apoptosis in keratinocytes is also heightened in psoriatic lesions, leading to the thickening of the epidermis [4]. Moreover, psoriatic phenotype could be induced by inhibiting apoptosis through the upregulation of anti-apoptotic genes and downregulation of pro-apoptotic genes [5], or proteins with anti-apoptotic effect like calcium/calmodulin-dependent protein kinase IV [6]. Surprisingly, mechanism researches revealed that psoriasis-like symptoms could be mitigated via promoting apoptosis [7, 8]. In clinical applications, strategies to alleviate psoriasis by inducting the apoptosis of keratinocytes have also been proposed successively like long-acting β2 adrenergic receptor [9] and UV radiation [10]. Due to the high correlation between apoptosis of keratinocytes and psoriasis prognosis, inducing apoptosis in keratinocytes is worth exploring as a potential therapeutic method.

G9A (gene name EHMT2), as one of the SUV family of H3K9 methyltransferases, was proved to have impact on prognosis of numerous cancers [11]. G9A and G9A-like-protein (GLP) interaction were proved to support multiple myeloma cell growth and survival by blocking the autophagy-associated apoptosis [12]. The induced apoptosis caused by inhibiting G9A in non-small-cell lung cancer A549 cells was also revealed [13]. A study found the IL-23 production in keratinocytes could be modulated by the methylation of H3K9 [14], demonstrating that G9A is connected to the inflammatory response such as psoriasis in keratinocytes. Nevertheless, the effect of G9A in regulating proliferation and apoptosis in keratinocytes during the progression of psoriasis is still poorly understood.

Mechanically, NF-κB is a significant mediator in the pathogenesis of psoriasis which links the hyperplasia and inhibited apoptosis of keratinocytes [15–17]. EDAR is one of the members forming a subgroup of the TNF-Receptor superfamily that can regulate apoptosis by utilizing a conserved intracellular signaling module called the death domain [18, 19]. Additionally, Li et al. demonstrated that EDAR associated via death domain (EDARADD) exhibited high levels of expression in head and neck squamous cell carcinoma tissues, and downregulation of EDARADD in tongue squamous cell carcinoma cells led to changes in apoptosis induction, proliferation suppression, and decreased expression of NF-κB [20]. A study conducted in hair follicles keratinocytes also revealed that inhibition of EDAR signaling could induce apoptosis during specific stage [21].

Based on researched all above, our closer investigation of the relationship between G9A function in keratinocytes and psoriasis now revealed that genetic ablation of *EHMT2* gene in keratinocytes provoked anti-proliferation effect and attenuated psoriatic dermatitis. Furthermore, through multiple experimental methods, we supposed that G9A could modulate activation of NF-κB pathway through affecting EDAR expression, and thus regulate apoptosis and proliferation of keratinocytes, which provides a new insight on G9A and apoptosis in psoriasis and holds a potential strategy on future psoriasis therapy.

## Materials and methods

### Animals

All BALB/c mice were purchased by Hunan SJA Laboratory Animal Co, Ltd. (Changsha, China). *Ehmt2*^fl/fl^ mice were constructed by GemPharmatech Co., Ltd. (Nanjing, China), which genetic background is C57BL/6 J. Mice with keratinocyte-restricted deletion of Ehmt2 were generated by crossing *Ehmt2*^fl/fl^ mice with K14-Cre transgenic mice. All mice were housed under specific pathogen-free conditions. Licenses for breeding and experiment were obtained from the Department of Laboratory Animals of Central South University (Changsha, China). In all animal experiments, controls were age- and gender-matched littermates.

### Mouse model of psoriasis and PASI score criteria

To induce psoriasiform skin lesion, we used IMQ cream (cat.19080639, Sichuan Med-Shine, China) for continuous topical application to ear or back skin of mice. The 6–8-week female BALB/c mice were randomly divided into 4 groups based on body weight: blank, IMQ, control and BIX01294 group. Except for the blank group, mice in other groups were subjected to a daily topical dose of 20 mg IMQ per ear for 3 consecutive days. At least 6 h after modelling per day, mice in the control group were treated with a topical application of 25 mg Vaseline ointment, and the BIX01294 group mice were topically applied with 25 mg Vaseline ointment containing 3% BIX01294 (cat. s8006, Selleck, USA). The 7–9-week female transgenic mice were treated with daily topical application of 62.5 mg of IMQ cream on the shaved dorsal skin for 5 consecutive days. The clinical Psoriasis Area and Severity Index (PASI) was used to monitor and evaluate the severity of skin inflammation. Erythema, thickness, and scaling of back skin were scored independently from 0 to 4 (0, none; 1, slight; 2, moderate; 3, marked; 4, severe). The cumulative score of each parameter was used as a measure of the severity of skin inflammation (scale 0–12). All procedures were approved by the Care and Use Committee of the Department of Laboratory Animals, Central South University.

### RNA isolation and qPCR

After fresh skin biopsy specimens were collected and flash-frozen in liquid nitrogen, tissues were fully broken with a homogenizer. Total RNA was extracted from cells or pretreated tissues using MagZol reagent (cat. R4801-01, Magen, Guangzhou, China). The synthesis of cDNA and qPCR was performed as previously described [22]. Related gene-specific primers are listed in Supplementary Table 1.

### RNA sequencing

RNA was extracted from HaCaT cells with knockdown EHMT2 or control. After the RNA sample was qualified, mRNA was enriched with magnetic beads with Oligo (dT). Then fragmentation buffer was added to break the mRNA into short fragments, using mRNA as the template to synthesize first-stranded cDNA using six-base random primer (random hexamers), and then buffer, dNTPs and DNA polymerase I and RNase H were added to synthesize second-stranded cDNA, after which the double-stranded cDNA was subsequently purified using AMPure XP beads. Purified double-stranded cDNA was subjected to end repair, plus A tail and ligation of sequencing joints, and then AMPure XP beads for selection of fragment size. Final PCR amplification was performed and the PCR products were purified using AMPure XP beads to obtain the final library. Once qualified, the libraries were sequenced using the Illumina high-throughput sequencing platform NovaSeq 6000. Analysis of the differential expression was performed using DESeq R package, and the differentially expressed genes were defined as log_2_ fold change > 1.0 and *P* value < 0.05. Annotation of differentially expressed genes was performed using David Database and Gene set enrichment analysis was performed with clusterProfiler R package.

### Western blotting

Cells or skin tissues pretreated with the homogenate were lysed into lysates in RIPA Lysis Buffer (cat. P0013C, Beyotime, Shanghai, China) containing protease and phosphatase inhibitors (cat. B14002, B15002, Bimake, USA). Lysates (20 μg protein/lane) were separated by SDS-PAGE and transferred to PVDF membranes (cat. IPVH00010, Millipore, USA). After blocking for 1 h at room temperature with 3% BSA in TBST, the PVDF membranes were incubated with specific primary antibodies for overnight at 4 °C. The membranes were washed with TBST at least 3 times and then incubated with appropriate secondary antibody at room temperature. Anti-G9A (cat. ab185050, Abcam, UK, 1:1000), anti-Caspase3 (cat. 9662, CST, USA, 1:1000), anti-BCL-2 (cat. 12789-1-AP, Proteintech, Wuhan, China, 1:2000), anti-BAX (cat. 50599-2-Ig, Proteintech, 1:2000), anti-GAPDH (cat. 60004-I-Ig, Proteintech, 1:3000), anti-P65 (cat. 8242 S, Abcam, 1:1000), anti-Pho-P65 (cat. 3033 S, CST, 1:1000), anti-NF-κB1 (cat. 13586 S, CST, 1:1000), anti-TRAF6 (cat. ab33915, Abcam, 1:2000), anti-EDAR (cat. ab137021, Abcam, 1:1000), anti-EDARADD (cat. A15950, Abclonal, Wuhan, China, 1:1000), anti-β-ACTIN (cat. sc-47778, Santa cruz, USA, 1:500), anti-TUBULIN (cat. 11224-1-AP, Proteintech, 1:2000), HRP Goat Anti-Mouse IgG (H + L) (cat.AS003, Abclonal), HRP Goat Anti-Rabbit IgG (H + L) (cat. AS014, Abclonal) were used as antibodies.

### Flow cytometry

To separate the dermis and epidermis, mouse skin lesions were digested in 1640 medium containing Dispase II (cat. D4693, Sigma, USA, 2 mg/ml) for at least 20 h at 4 °C. And then dermis was treated with 1640 medium containing collagen IV (cat. V900893, Sigma, 2 mg/ml) for 1 h at 37 °C and epidermis was digested in 0.05% trypsin-EDTA (cat. C0201, Beyotime,) for 10 min at 37 °C to obtain single-cell suspensions of skin tissues. To detect the expression of IL-17A and IFN-γ in skin, cell suspensions were cultured and incubated with Cell Stimulation Cocktail (plus protein transport inhibitors) (cat. 00-4975-93, eBioscience, USA) for 6 h at 37 °C. After stimulation, the cells were washed and stained with a fixable living/dead cells dye (cat. 564997, Horizon™ Fixable Viability Stain 700, BD, USA) for 10 min on ice. After blocked with trustain fcX anti-mouse CD16/32 (cat.101320, Biolegend, USA), cells were surface-stained with APC/CY7-conjugated anti-CD45 (cat. 103116, Biolegend), BB700-conjugated anti-CD3e (cat. 566494, BD), BV421-conjugated anti-CD4 (cat. 562891, BD), BV711-conjugated anti-TCRβ (cat. 109243, Biolegend) and FITC-conjugated anti-TCRγ (cat. 107503, Biolegend) for 40 min on ice. And cells were washed twice and permeated with True-Nuclear Transcription Factor Buffer Set for 12 h at 4 °C (cat. 424401, Biolegend). The cells were stained with PE-conjugated anti-IL-17A (cat. 559502, BD) and APC-conjugated anti-IFN-γ (cat. 505810, Biolegend) for 1 h at room temperature. Data were obtained using BD FACS LSRFortessa flow cytometer and analyzed using the FlowJo software.

### Histological analysis

Frozen or paraffin-embedded skin specimens were sectioned to consecutive levels of thickness. Sections were stained with hematoxylin and eosin (H&E) according to standard procedures. For immunostaining analyses of tissues, the sections were deparaffinized and stained with antibodies against G9A (cat. ab185050, Abcam, 1:500), Ki67 (cat. ab16667, Abcam, 1:200), PCNA (cat. ab15497, Abcam, 1:200).

### Cell culture & in vitro transfection & inhibitor experiments

The human immortal keratinocyte cell line HaCaT was purchased from ATCC and cultured in a medium 1640 supplement with 10% fetal bovine serum. To obtain primary normal human epidermal keratinocytes (NHEK), discarded adult foreskin biopsy tissues were collected from the Department of Urology, Xiangya Hospital, Central South University. After subcutaneous adipose tissues were removed, the foreskin tissues were digested in medium 1640 containing Dispase II (2 mg/ml) for overnight at 4 °C. Next, the epidermis was isolated and then digested into a single cell suspension with 0.05% trypsin-EDTA. Cells were seeded in 100 mm cell dishes at the appropriate density. NHEK were cultured in the serum-free medium supplement with indicated growth factors (cat. 192060, Lonza, CH) and subcultured according to the cell fusion. For RNA interference, HaCaT cells were transfected with siRNA (purchased from Genepharma, Shanghai, China) against RELA, NFKB1 or vehicle using Lipofectamine 2000 Transfection Reagent (cat. 11668019, Thermo Fisher, USA) for 48 h. To knockdown of EHMT2 in keratinocytes, 293 T cells were firstly transfected with shRNA of EHMT2 (purchased from Beijing Syngentech Co., Ltd. China) or negative control together with pspAX2, PMD2.G for overnight. After 2–3 days for transfection, supernatant of lentivirus was collected and concentrated with a lentiviral concentration kit (cat. BG20101L, Beijing Syngentech). Keratinocytes were then transfected with concentrated lentivirus to knock down the target gene. For inhibitor experiments, keratinocytes were treated with BIX01294 (dissolved in water, 2.5, 5,10 μM), 5 μM QNZ (cat. S4902, Selleck) for indicated times.

### CCK-8 assay

The ability of cell proliferation was determined by cell counting kit-8 (CCK-8) assay (cat. B34302, Selleck). Briefly, cells were planted in 96-well plates (2500 cells/well) and examined every 24 h, 10 µl of CCK8 solution and 100 µl medium were added into each well and measured spectrophotometrically at 450 nm after 2 h of incubation.

### Colony formation assay

To assess differences in colony formation ability, cells with different treatments were seeded into 6-well plates (1500 cells/well) and cultured for 1–2 weeks according to the size of the colony formed. Colonies were washed twice with DPBS, then fixed with 4% paraformaldehyde for 15 min and stained with Crystal Violet Staining Solution (cat. C0121, Beyotime) for 20 min, and finally wash with DPBS and dried. The image of colonies was scanned by EPSON scanner and the number of colonies was counted using ImageJ software.

### Cell apoptosis

To analyze the apoptosis rate of cells, Annexin V-Alexa Fluor 647/PI Apoptosis Detection Kit (cat. FXP023a-500U, 4 A BIOTECH, Suzhou, China) was used according to the manufacturer’s instructions. Cells were collected, washed with pre-cold DPBS and resuspended in 1× binding buffer. Next, cells were stained with 5 µl diluted Annexin V-AF647 in the dark for 5 min and then added and incubated with 10 µl propidium iodide for 15 min at room temperature. Data were obtained using BD FACS LSRFortessa flow cytometer.

### Statistical analysis

Data are presented as Mean ± SEM and analyzed using the GraphPad Prism with the paired Student’s *t*-test, unpaired Student’s *t*-test, One-way ANOVA or Two-way ANOVA. All experiments were repeated three or more times. *P*-value less than 0.05 was considered significant, statistical significance was defined as *P < 0.05, **P < 0.01, ***P < 0.001, ****P < 0.0001.

## Results

### Keratinocyte-specific depletion of *Ehmt2* protected against IMQ-induced psoriasis

**Fig. 1:**
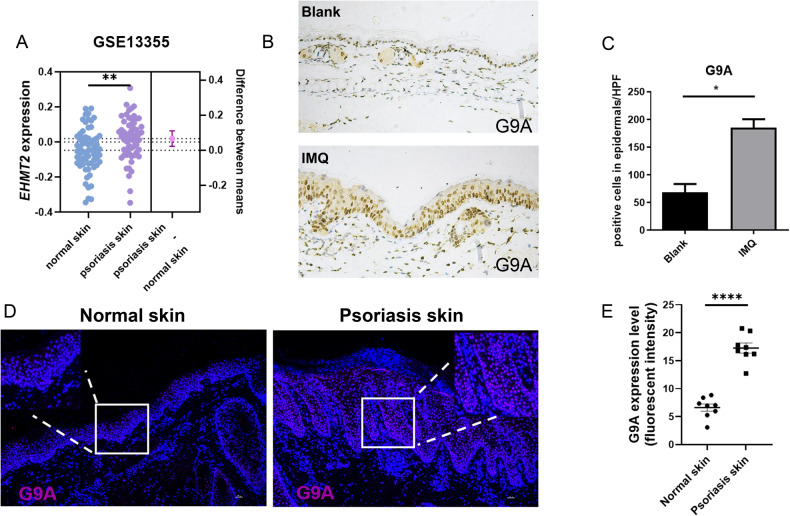
G9A expression in human and mouse skin. **A** Comparison of *EHMT2* mRNA levels in normal human skin (*n* = 64) and psoriasis lesion skin (*n* = 58) from the Gene Expression Omnibus (GEO) Database (accession number: GSE 13355). **B** Immunohistochemical staining of G9a in skin sections derived from wildtype mice (up, 200×) and IMQ-induced mice (down, 200×). **C** Quantification of G9a positive cell in epidermis. **D** Normal human skin (left, 100×) and psoriasis lesion skin (right, 100×) immune-stained for G9A. Scale bars, 100 µm. **E** Quantification of G9A expression in epidermis keratinocyte. The values are presented as the mean ± SEM. *p < 0.05, **p < 0.01 and ****p < 0.0001. (Unpaired Student’s *t* test).

Previously, it was shown that EHMT2, as a H3K9me1 and H3K9me2 catalyzing enzyme, owns a crucial potential on regulating cell proliferation and was always seemed as a tumor promoting gene in various cancer diseases [23–25]. However, the characteristic of EHMT2 on modifying prognosis of psoriasis remains undetermined. Thus, in order to clarify the correlation between EHMT2 and psoriasis, starting with the clinical phenomenon, we found that the expression of *EHMT2* was upregulated in lesion skin samples derived from psoriasis patients compared with healthy human skin samples (Fig. 1A), according to GEO database. We furtherly confirmed the elevated mRNA expression of EHMT2 in psoriatic lesions contrast to normal skin (Fig. S1E). However, we did not observe the similar phenomenon in other inflammatory skin diseases such as atopic dermatitis (AD), systemic lupus erythematosus (SLE), and acne (Fig. S1A–C) in accordance with GEO database. We demonstrated that G9A expression was elevated after stimulation with topical IMQ cream on mouse model (Fig. 1B, C). To further analyze the G9A protein expression profile in clinical psoriasis disease, we conducted immunofluorescence staining on skin biopsy specimens of psoriasis patients and healthy controls. In healthy skin samples, G9A was weakly expressed in the nuclear of epidermal keratinocytes of the basal layer, on the contrary, it was obviously observed in the epidermal keratinocytes from psoriasis skin lesions (Fig. 1D, E). Meanwhile, G9A was also strongly observed in epidermis of psoriatic lesions by IHC (Fig. S1E, F). Above-mentioned evident disclosed that excessive expression of G9A is intensively linked with psoriasis.

**Fig. 2:**
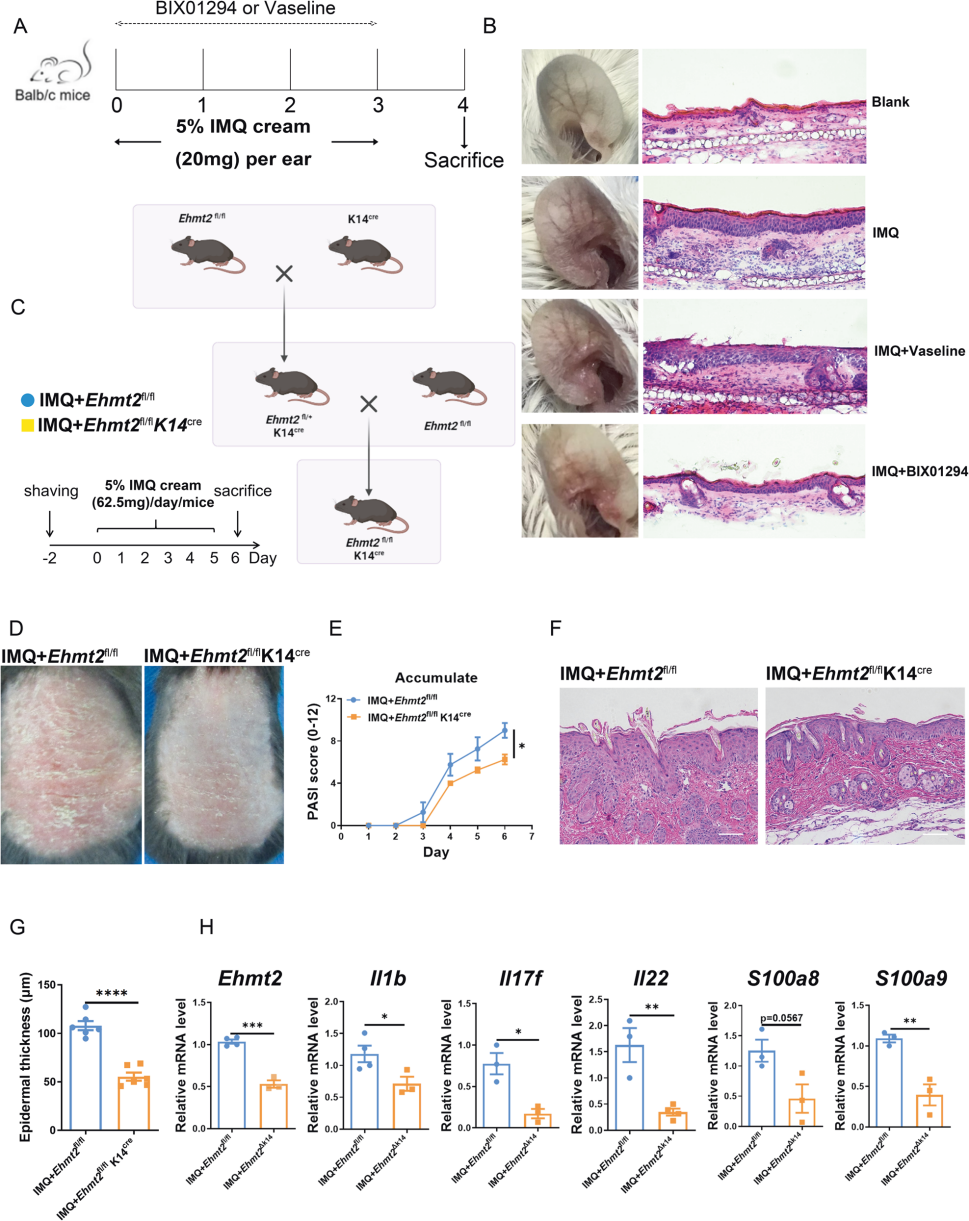
Keratinocyte specific knock-out of EHMT2 protects against IMQ induced psoriasis-like dermatitis. **A** Psoriasiform dermatitis was induced on the ear skin of BALB/c mice by topical IMQ cream application (20 mg/ear) for 3 consecutive days, during which BIX-01294 ointment (25 mg/ear) or equivalent volume of Vaseline ointment used as control was administered topically (*n* = 5). **B** Representative images of mouse ear on day 4. Pathological sections of lesion skin in each group were listed on the right (200×). **C** Breeding scheme for the generation of keratinocyte (K14)-specific Ehmt2 knock-out mice. Psoriasis-like skin inflammation was induced by topical application of IMQ cream daily for 5 consecutive days. **D** Representative photos of back skin from *Ehmt2*^fl/fl^ and *Ehmt2*^fl/fl^ K14^cre^ mouse (*n* = 6). **E** Daily PASI score indicated by back skin scaling (0–4), erythema (0–4), and thickness (0–4) in IMQ-treated mice. **F** Representative H&E staining of back skin. Scale bars, 100 µm. **G** Epidermal thickness was measured. **H** mRNA levels of inflammatory cytokines in back skin were examined by qPCR. Each dot represented an individual mouse. The values are presented as the mean ± SEM. *p < 0.05, **p < 0.01, ***p < 0.001 and ****p < 0.0001. (Unpaired Student’s *t* test or two-way ANOVA).

To investigate whether inhibiting function of G9A exerts anti-psoriatic effects, we prescribed G9A inhibitor BIX01294 as topical ointment treatment for mouse model of psoriasis. Psoriasis was induced by topical IMQ application for 3 consecutive days (Fig. 2A). On the 4th day, the ears of IMQ-treated mice showed markedly thickened, erythematous compared with the blank group (Fig. 2B), which was dramatically dampened in BIX group contrasted with Vaseline group seemed as negative control. In order to uncover the explicit function of EHMT2 on psoriasis, we generated keratinocyte specific knock-out of *Ehmt2* mouse by means of crossing mice with loxP-flanked *Ehmt2* alleles (*Ehmt2*^fl/fl^) with Keratin14-Cre (K14) mice to delete *Ehmt2* in keratinocytes, and testified its efficiency (Fig. 2C, Fig. S2A, B). We did not observe a significant distinction between the thickness and structure of epidermis on two kinds of adult mice before any interference (Fig. S2C–E). After conducting 5-day IMQ treatment, *Ehmt2*^fl/fl^ K14^cre^ mice showed alleviated psoriasis-like features, including lessened erythema and scale (Fig. 2C–E) without alternation in the spleen index or weight changes (Fig. S3B–D). Histological analysis of skin samples derived from *Ehmt2*^fl/fl^K14^cre^ mice revealed a thinner cornified layer (less hyperkeratosis) and restricted epidermal hyperplasia (limited acanthosis), while compared with *Ehmt2*^fl/fl^ mice (Fig. 2F, G). Consistent with previous results, the mRNA levels of inflammatory related cytokines were markedly reduced in *Ehmt2*^△K14^ mice (Fig. 2H). Besides, we also detected the expression levels of keratinocyte differentiation related genes containing involucrin (Ivl), filaggrin (Flg), keratin 16 (Krt16), keratin 6a (Krt6a), and keratin 10 (Krt10), but there was no significant difference in two groups (Fig. S3A). On the basics of showed proof, we illustrated that diminishing the expression of EHMT2 or inhibiting the function of EHMT2 is correlated with ameliorated symptom in psoriasis.

### Flow cytometry elucidated attenuated autoimmune response in keratinocyte specific Ehmt2-KO mice after IMQ treatment

**Fig. 3:**
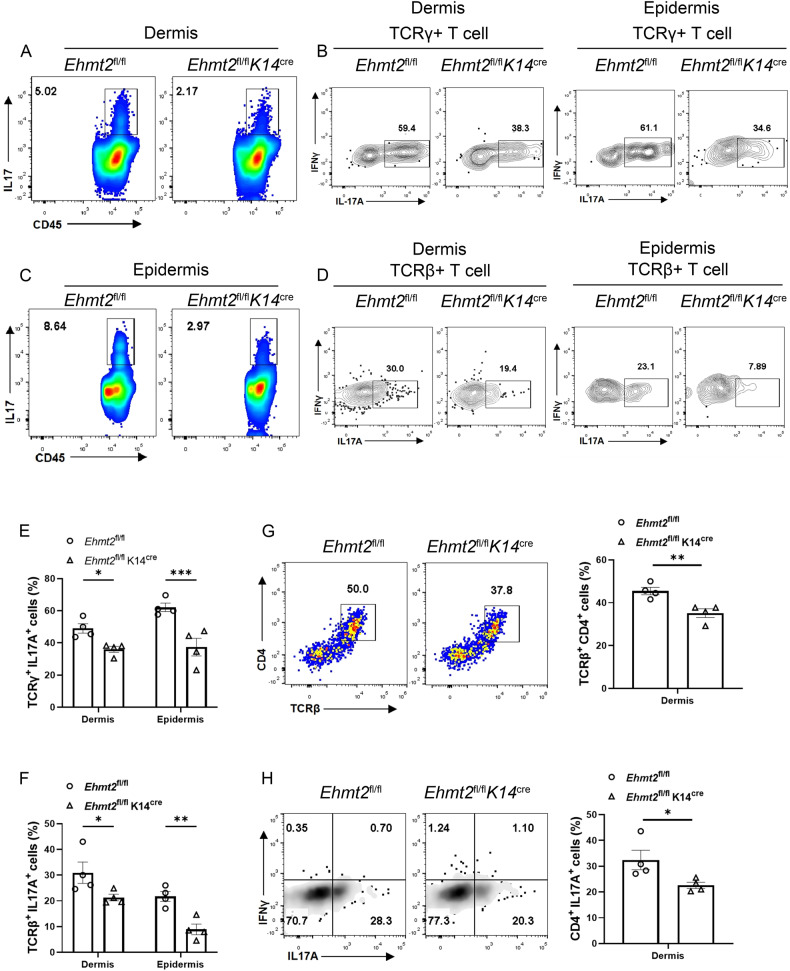
Flow cytometric analysis of T cell subcluster in epidermis and dermis. Representative flow plots and subpopulations of CD45^+^ IL17A^+^ cells in dermis (**A**) and epidermis (**C**). Ratio of IL17-producing γδ T cells (TCRγ^+^ IL17A^+^) (**B**) and IL17-producing αβ T cells (TCRβ^+^ IL17A^+^) (**D**) are shown. **E**, **F** Quantification. **G** Population of CD4^+^ T cells (TCRβ^+^ CD4^+^) in dermis. **H** Percentage of IL17A^+^ and IFNγ^+^ cells gated on CD4^+^ T cells in dermis. The values are presented as the mean ± SEM. *p < 0.05, **p < 0.01 and ***p < 0.001. (Unpaired Student’s *t* test).

After utilizing flow cytometric analysis on the back skin lesional tissues of psoriasis mouse model (Fig. S4), we showed decreases in the numbers of CD45^+^ IL17^+^ cells in dermis and epidermis (Fig. 3A, C). Further analysis revealed that IL-17A is derived from both infiltrating αβ and γδ T cells in control and *Ehmt2*^fl/fl^ K14^cre^ mice, while the ratio of IL-17A-expressing γδ T and αβ T cells was suppressed in IMQ-treated K14-*Ehmt2*–KO mice (Fig. 3B, D, E, F). We also measured IFNγ producing ability on T cells and found no distinction after conditional knocking out *Ehmt2* (statistic data were not shown). Due to Th17 cells is a variety of widely-acknowledged pathogenic factor in psoriasis [26, 27], we examined both ratio of CD4^+^ T cells in dermis and percentage of Th17 cells in dermis, and interestingly, we delineated that EHMT2 knock out mouse accompanied with deficient capacity on Th17 cells polarization and reduced infiltrated CD4^+^ T cells (Fig. 3G, H), which brought constrained inflammation during psoriasis-like dermatitis.

### EHMT2 accommodated keratinocyte proliferation and apoptosis

**Fig. 4:**
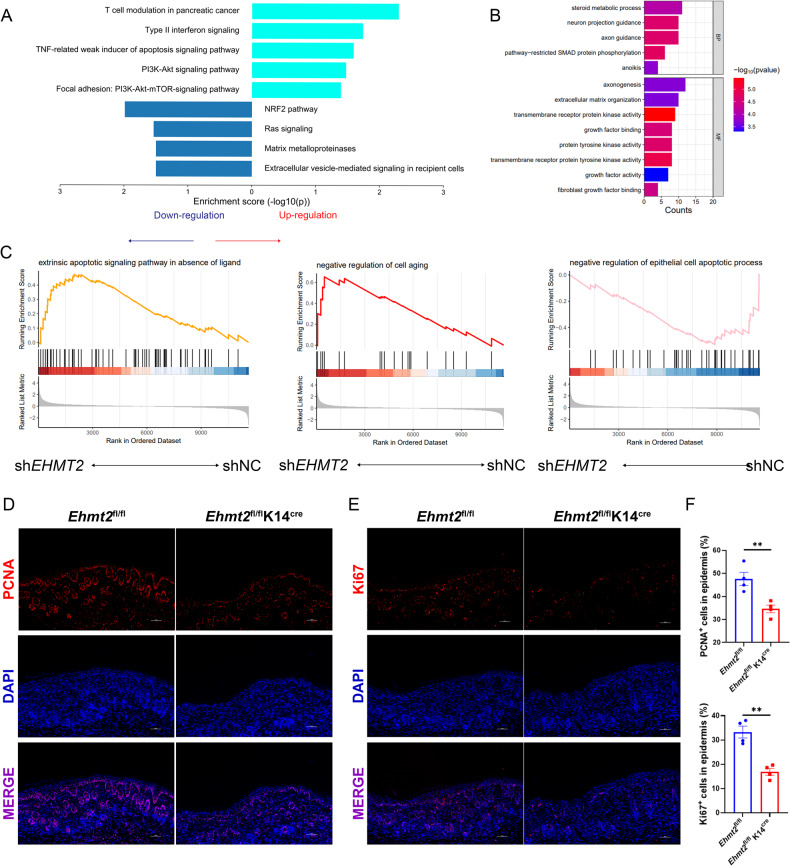
EHMT2 administrates apoptosis and cell cycle of keratinocyte. **A** WikiPathway enrichment in sh*EHMT2* HaCaT cells compared with shNC. **B** Gene Ontology enrichment displayed down-regulated pathway in sh*EHMT2* vs shNC cells. **C** Gene set enrichment analysis showed that apoptosis related function as well as cell cycle progression was significantly regulated by EHMT2. Immunofluorescence of PCNA (**D**) and Ki67 (**E**) expression. **F** Quantification of PCNA and Ki67 expression. Scale bar, 100 µm. The values are presented as the mean ± SEM. **p < 0.01. (Unpaired Student’s *t* test).

Relying on anterior research [14] and our experiment in vitro (Figs. S5, 6), the phenotype that proinflammatory cytokines were enhanced in *EHMT2* knocking down keratinocytes was not able to explain the reason why eliminating EHMT2 elicited the protective ability on psoriatic dermatitis. Therefore, to further clarify the underlying mechanism, we performed RNA-seq analysis on sh*EHMT2* and shNC HaCaT. Pathway enrichment analysis discovered that upregulated significant different genes involved in TNF related weak inducer of apoptosis signaling pathway, on the other hand, downregulated genes enriched in Ras signaling, which plays a pivotal role in cell proliferation (Fig. 4A). Gene Ontology enrichment exhibited several cell growth related functions (Fig. 4B). These findings suggested that knocking down *EHMT2* resulted in anti-proliferation and pro-apoptosis function in HaCaT cells. Furthermore, Gene set enrichment analysis on Gene Ontology further demonstrated this hypothesis owing to its interrelated with cell apoptosis (Fig. 4C). Meanwhile, we corroborated that keratinocyte specific knocking out of EHMT2 restrained cell proliferation in mouse skin lesion after applying IMQ smearing by conducting immunofluorescence on Ki67 and PCNA considered as the marker of cell proliferation, in which Ki67^+^ and PCNA^+^ cell counts were significantly diminished compared with IMQ-treated *Ehmt2*^fl/fl^ mice (Fig. 4D-F).

**Fig. 5:**
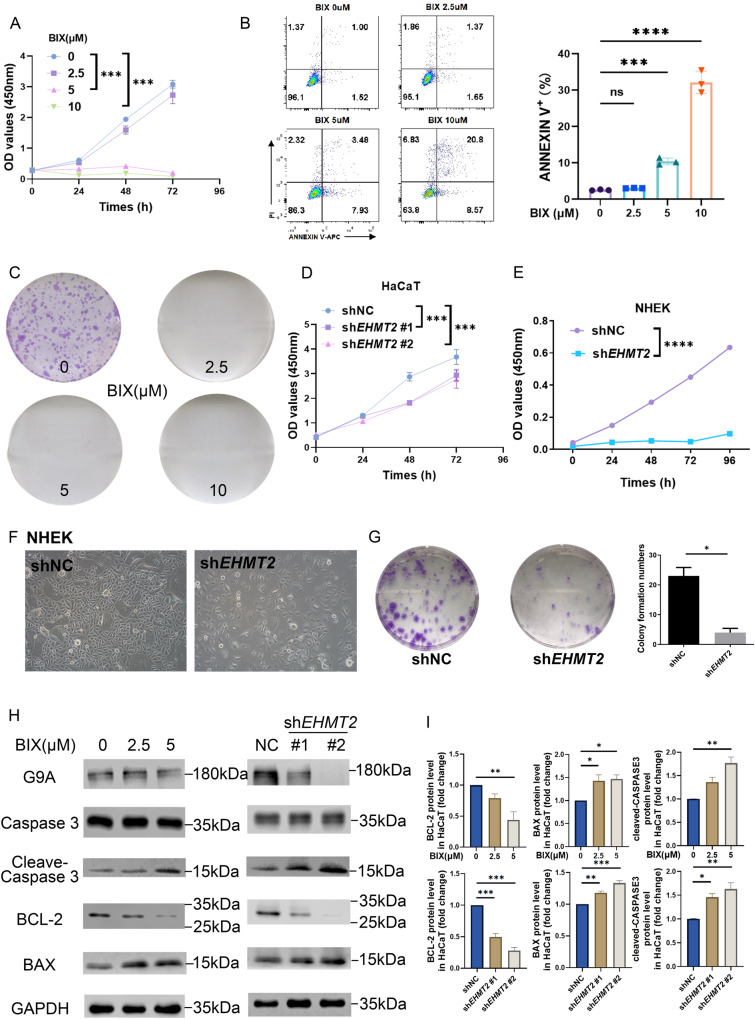
Suppression of keratinocyte proliferation and regulation of keratinocyte apoptosis by EHMT2. **A** Proliferation of HaCaT cells treated with a serial dose of BIX01294 (BIX) at different time period was detected by CCK-8 assay. **B** Apoptosis of HaCaT cells after treatment with a serial dose of BIX01294 for 24 h was examined through flow cytometry. **C** Colony formation assay of HaCaT cells after the indicated treatment. The viability of HaCaT (**D**) or NHEK (**E**) cells transfected with either *EHMT2* shRNA or control shRNA was evaluated at different time period by CCK-8 assay. **F** Cell morphology of NHEK was examined by microscopy. **G** Colony formation assay of NHEK cells after transfected with respective shRNA. **H** Western blot analysis of the indicated proteins in shNC, sh*EHMT2* HaCaT cells and BIX treated HaCaT cells. **I** Quantification. The values are presented as the mean ± SEM. *p < 0.05, **p < 0.01, ***p < 0.001 and ****p < 0.0001. (Unpaired Student’s *t* test or two-way ANOVA).

The anti-proliferation effect of executing G9A inhibitor interference and inhibiting expression of EHMT2 on keratinocyte was attested through CCK-8 in vitro (Fig. 5A, D, E). We further evaluated cell apoptosis in HaCaT after giving BIX treatment using flow cytometry (Fig. 5B), which turned out that suppressing the function of G9A caused keratinocyte apoptosis. In addition, to solidly investigate the antiproliferative effect of inhibiting G9A, a colony formation assay was performed on BIX dose-related treatment in HaCaT cells and *EHMT2* knocking down NHEK (Fig. 5C, G), and these results showed that impaired function on G9A significantly suppressed cell proliferation. According to previous experiments, the 5 µM BIX treatment owned an adequate inhibition on keratinocyte proliferation, thus we conducted further study based on this concentration. The immunoblotting results further revealed correlated changes in the expression levels of these apoptosis markers including Caspase3, BAX, Bcl-2, during BIX treating and EHMT2 depleting (Fig. 5H, I), which was unseparated interconnected with the flow cytometry data on apoptosis.

### EHMT2 generated keratinocyte apoptosis via the EDAR/NF-κB axis

**Fig. 6:**
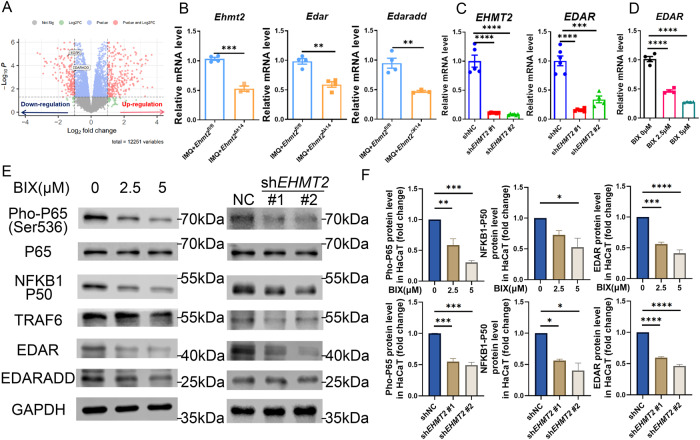
EHMT2 regulates EDAR expression and NF-κB signaling. **A** Volcano map of significant different gene in sh*EHMT2* vs shNC. **B** qPCR of *Ehmt2*, *Edar, Edaradd* in lesional skin from IMQ-treated *Ehmt2*^fl/fl^ and *Ehmt2*^fl/fl^ K14^cre^ mice. Each dot represented an individual mouse. **C**, **D** qPCR of *EHMT2*, *EDAR* in HaCaT. **E** Immunoblot of EDAR- NF-κB signal related protein in HaCaT transduced with *EHMT2* shRNA or treated with BIX. **F** Quantification of protein expression. The values are presented as the mean ± SEM. *p < 0.05, **p < 0.01, ***p < 0.001 and ****p < 0.0001. (One-way ANOVA).

EHMT2 has been reported to modulate cell cycle in cancer cells by specifically regulating the expression of certain genes or the activity of signaling pathways [13, 28]. Therefore, we reanalyzed the RNA-seq data of sh*EHMT2* vs shNC HaCaT cells and surprisingly found out a downregulated gene named EDAR (Fig. 6A). EDAR, as a member of the tumor necrosis factor (TNF) family, is widely assumed as the upstream of NF-κB in accordance to KEGG pathway database termed NF-kappa B signaling pathway (map04064) and past research [29]. In addition, we found that the mRNA level of *Edar* was lower in the epidermis of *Ehmt2*^fl/fl^ K14^cre^ mice compared with *Ehmt2*^fl/fl^ mice after IMQ induction (Fig. 6B), and verified that EHMT2 did monitor the transcription expression of EDAR through qPCR in vitro (Fig. 6C, D). After BIX administration or sh*EHMT2* transfection, we quantified the protein expression associated with EDAR pathway specifically including EDAR, EDARADD, TRAF6, pho-P65 and P50, in which the expression of NF-κB targets were dramatically inhibited, suggesting that EHMT2 can markedly regulate EDAR/NF-κB signaling activity (Fig. 6E, F).

**Fig. 7:**
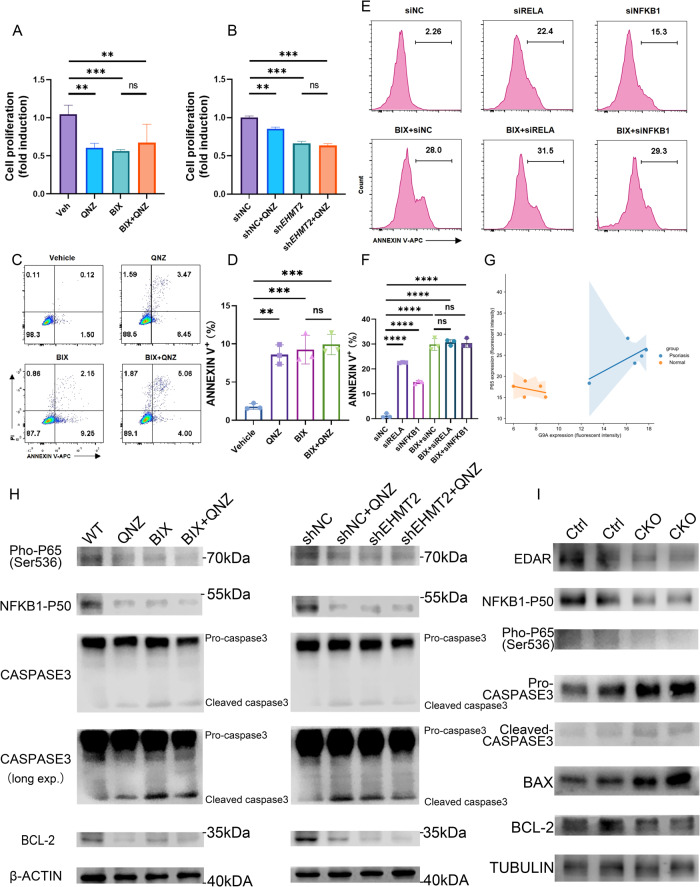
EHMT2 governs keratinocyte apoptosis through NF-κB family. **A** The viability of HaCaT cells were examined after treating with either QNZ (5 µM) alone or BIX (5 µM) alone, or in combination with QNZ (5 µM) & BIX (5 µM). **B** The viability of shNC and sh*EHMT2* HaCaT cells were evaluated after treating with or without QNZ (5 µM). **C**, **D** Apoptosis of HaCaT cells were analyzed after treatment with either QNZ (5 µM) alone or BIX (5 µM) alone, or in combination with QNZ (5 µM) & BIX (5 µM). **E**, **F** Apoptosis of HaCaT cells were determined through flow cytometry after being transfected with siRNA (siNFKB1/siRELA/siNC) or treated with BIX alone (5 µM) or in combination for 24 h. **G** Correlation between G9a protein expression and P65 protein expression in psoriasis skin lesion through conducting immunofluorescence. **H** Activation of NF-κB signaling and promotion of apoptosis was determined by immunoblotting. **I** Immunoblotting of indicated proteins in the epidermis from 3-day IMQ induced control mice and cKO mice. Data are presented as the means ± SEM. **p < 0.01, ***p < 0.001 and ****p < 0.0001. ns not significant. (One-way ANOVA).

Considering the ability of NF-κB family on administrating cell cycle and apoptosis [30], we hypothesized whether G9A monitoring cell proliferation through NF-κB signaling pathway. To verify this assumption, we first detected the linkage between expression of G9A and NF-κB p65 in clinical psoriasis skin lesion by conducting immunofluorescence, which turned out a positive correlation in psoriasis sample (Fig. 7G). Furthermore, we treated HaCaT cells with either QNZ (NF-κB inhibitor) alone or BIX alone, or in combination with QNZ & BIX. Treating QNZ alone and BIX alone both exhibited a remarkable competence on prohibiting cell proliferation and resulting in cell apoptosis (Fig. 7A, C, D). Immunoblot experiment ulteriorly validated that after QNZ and BIX sole application, expression of apoptosis associated marker protein comprising BAX, cleaved-caspase3 all evaluated, and anti-apoptosis protein termed Bcl-2 declined (Fig. 7H). In contrast, employing QNZ and BIX in combination was not capable of bringing extra effect on restraining cell proliferation or eliciting cell apoptosis while compared with either QNZ group or BIX group (Fig. 7A, C, D). This interesting phenomenon was also observed in immunoblot experiment while analyzing the expression of protein (Fig. 7H). All these evidences pointed to an explanation that due to inactivating G9A yielded attenuated activation of NF-κB, we could not observe an overlay effect on resulting in cell apoptosis. Moreover, we extended this conclusion to *EHMT2* knocking down HaCaT cells. In brief, knocking down EHMT2 owned sufficient potential on inducing apoptosis, while additional QNZ interference would not play an extra part in this process (Fig. 7B, H). Owing to forward results on G9A regulating NF-κB p65 and NF-κB p50 activation (Fig. 6E), we performed apoptosis assay on HaCaT cells transfected with siRNA (siNFKB1 or siRELA) and treated with BIX, and revealed that treating BIX after silencing P65 or NFKB1 would not cause added cell apoptosis (Fig. 7E, F). Ultimately, in vivo aspect, compared with control mice, western blot analysis on the epidermis derived from *Ehmt2*^fl/fl^ K14^cre^ mice uncovered a systematic clarification on downstream signaling pathway and illustrated our previous inference (Fig. 7I). Taken together, our research identified that G9A administrated cell apoptosis through EDAR-NF-κB pathway.

## Discussion

G9A and GLP form a heteromeric complex responsible for H3K9me1 and H3K9me2, contributing to gene silencing and heterochromatin formation [31]. Recent studies highlight their significant roles in various biological processes, including tumor development [11]. Mechanistically, the growth and metastasis of breast cancer necessitate the presence of G9A, which fosters cell proliferation [32, 33]. In lung carcinoma, G9A participates in the activation of WNT signaling by suppressing relating inhibitors like APC2, DKK1, and WIFI, which ultimately promotes cell proliferation [34]. When exposed to Chaetocin, a H3K9me inhibitor, lung cancer cells also exhibit increased apoptosis with decreased H3K9me3 levels [35]. Rich evidence about the regulating effect of G9A in tumor cells are demonstrated, but the role of G9A in controlling the proliferation and apoptosis of keratinocytes during the advancement of psoriasis remains inadequately comprehended.

Li, Hui et al. uncovered that knocking-out N-WASP caused limited H3K9 di-methylation due to accelerating protein degradation of G9A and GLP in keratinocytes, upregulating the IL-23 expression [14], which corresponds to a part of our findings that depleting G9A upregulated proinflammatory cytokines in HaCaT cell line and NHEK (Supplementary Figs. 5, 6). However, the researchers did not investigate the direct correlation between G9A that owns multiple function except for acting as H3K9 methyltransferase and psoriasis phenotype in vivo. On the contrary, we demonstrated a positive correlation between G9A and the prognosis of psoriasis from clinical data and animal models, in which downregulating G9A accompanied with ameliorated psoriasis phenotype (Figs. 1, 2). Even though the pro-inflammatory effect of depleting G9A in vitro is inappropriate to explain its impact on the psoriasis development, disorder in inflammatory response is just one of the dysfunctions in pathogenic mechanism of psoriasis. Several factors including over-proliferation of keratinocyte should be considered, so that we can figure out the full picture on psoriasis (Figs. 5–7). Surprisingly, multiple studies supported our hypothesis that G9A may promote keratinocyte proliferation and inhibit cell apoptosis, particularly in Tine et al.’s study, which mentioned that the ability of N-WASP seemed as the upstream of G9A, according to Li, Hui’s research, to promote keratinocyte proliferation brought an adverse expectation on function of N-WASP in psoriasis. In conclusion, our study brought a proper conception on psoriasis and G9A.

NF-κB, acted as a transcription factor of Bcl-2, is a crucial regulatory pathway in cellular proliferation, differentiation, and apoptosis in the pathogenesis of psoriasis [15, 36]. In our study, we discovered that the inhibition of keratinocyte proliferation and more importantly, the promotion of apoptosis, is mediated by inhibiting NF-κB pathway with QNZ, siRELA and siNFKB1 (Fig. 7A–E). Existing studies also found the Piasy-Trim32 interaction induced keratinocyte apoptosis is mediated by NF-κB [37], and NF-κB has also been found to be associated with apoptosis in other cell types, such as osteoarthritis [38], non-alcoholic steatohepatitis macrophages [39], colon cancer [40] and ovarian tumors [41], which is consistent with our findings. Meanwhile, by downregulating G9A, our results suggested that the downstream of NF-κB pathway could be modulated by G9A, thus affected apoptosis subsequently, as the combined effect of QNZ and BIX did not exhibit significant difference compared with their respective effects when used alone (Fig. 7A–F, H). As previous research, Garcin, et al. provide evidence that the manipulation of EDAR signaling brought about alterations in multiple aspects in the process of adult skin wound healing, turning out that EDAR anticipated in cell proliferation [42]. The EDAR-mediated NF-κB activation is also proved, in which TRAF6 is an essential factor [43]. Therefore, based on the fact that G9A regulated mRNA and protein expression of EDAR (Fig. 6A–E), we confirmed the role of the G9A-EDAR-NF-κB signaling pathway in regulating the apoptosis in keratinocytes.

Previous studies have demonstrated that the resistance of keratinocytes to apoptosis is a crucial pathophysiological mechanism in psoriasis [4, 44], with numerous approaches promoting keratinocyte apoptosis are proved to ameliorate psoriasis like the use of long-acting β2 adrenergic receptor agonist [9], oxymatrine [45], overexpression of miR-383 [7] and downregulation of miR-155 [46]. According to our results, inhibiting G9A with BIX or directly knocking down the EHMT2 gene significantly increased the expression of apoptosis-related proteins caspase3 and BAX and decreased Bcl-2 expression, thus led to a notable decrease in cell growth of HaCaT and NHEK (Fig. 5). These findings further confirmed that G9A might be a potential target on curing psoriasis in the future through regulating KC proliferation and apoptosis.

Although we have proved that knocking down or inhibiting G9A function can downregulate EDAR expression through multiple experiments and sequencing data, we did not thoroughly investigate the mechanism by which G9A regulates EDAR expression on the aspect of epigenetic, but this may provide a direction for future research in this area. In summary, our data validated the unidentified role for G9A-EDAR-NF-κB pathway affecting psoriasis development by regulating the apoptosis of keratinocytes.

### Supplementary information

Supplementary Figure and Table Legends

Figure S1

Figure S2

Figure S3

Figure S4

Figure S5

Figure S6

Supplementary Table 1

checklist

Original Data File

Authorship Change Approval

## Data Availability

Data presented and analyzed in this study are available from the corresponding author on reasonable request.
