# Supplementary material for: Depletion of G9A attenuates imiquimod-induced psoriatic dermatitis via targeting EDAR-NF-κB signaling in keratinocyte
Source: Cell Death Dis. 2023 Sep 22;14(9):627. doi: 10.1038/s41419-023-06134-y (PMC10517171; doi:10.1038/s41419-023-06134-y)
Supplement: Supplementary file 1 — Supplementary Figure and Table Legends [file 41419_2023_6134_MOESM1_ESM.docx]

**Supplementary Fig. 1 G9A expression in different inflammatory skin diseases**

**(**A) Comparison of *EHMT2* mRNA levels in atopic dermatitis lesional skin (*n* = 8) and nonlesional skin (*n*= 8) or normal human skin (*n* = 6) from the GEO Database (accession number: GSE 182740). (Left: paired Student’s *t* test, right: Unpaired Student’s *t* test) (B) Comparison of *EHMT2* mRNA levels in acne lesional skin (*n* = 6) and nonlesional skin (*n* = 6) or normal human skin (*n* = 6) from the GEO Database (accession number: GSE 6475). (Left: paired Student’s *t* test, right: Unpaired Student’s *t* test) (C) Comparison of *EHMT2* mRNA levels in normal human skin (*n* = 7) and lesion skin (*n* = 7) from patients with SLE through the GEO Database (accession number: GSE 124939). (Unpaired Student’s *t* test) (D) The mRNA expression of EHMT2 in psoriatic lesions (*n*=4) and normal skin tissues (*n*=4) through qPCR (Unpaired Student’s *t* test) (E) Immunohistochemical staining of G9A in skin sections derived from normal skin (left, 100×) and psoriasis skin (right, 100×). (F) Quantification of G9A positive cell in epidermis. (Unpaired Student’s *t* test)

**Supplementary Fig. 2 Epidermal morphology and thickness of ear and back skin in mice**

(A)Ubiquitous expression of G9a in the skin of *Ehmt2*^fl/fl^ mouse (up, 100×), which is restrictedly absent in the epidermis of *Ehmt2*^fl/fl^ K14^cre^ mouse (down, 100×), rather than interfering dermis cell. (B) Genotyping results for *Ehmt2*^fl/fl^ and *Ehmt2*^fl/fl^ K14^cre^ mouse. (C) H&E-stained ear tissue sections from *Ehmt2*^fl/fl^ and *Ehmt2*^fl/fl^ K14^cre^ mouse. (D) Quantification of epidermal thickness of ear. (Unpaired Student’s *t* test) (E) High resolution image of H&E-stained back skin sections from *Ehmt2*^fl/fl^ and *Ehmt2*^fl/fl^ K14^cre^ mouse (200×).

**Supplementary Fig. 3 Keratinocyte specific knock-out of Ehmt2 had no effects on epidermal differentiation, body weight, spleen, and spleen index of mice**

(A) The mRNA levels of keratinocyte differentiation related genes in *Ehmt2*^fl/fl^ and *Ehmt2*^fl/fl^ K14^cre^ mouse. (Unpaired Student’s *t* test) (B) Body weight changes in *Ehmt2*^fl/fl^ and *Ehmt2*^fl/fl^ K14^cre^ mouse during modeling induced by IMQ. (C) Gross view of the spleen in *Ehmt2*^fl/fl^ and *Ehmt2*^fl/fl^ K14^cre^ mouse at the end point of the study. (D) The index of spleen in *Ehmt2*^fl/fl^ and *Ehmt2*^fl/fl^ K14^cre^ mouse at the end point of the study. (Unpaired Student’s *t* test)

**Supplementary Fig. 4 Flow gating strategy in mouse skin tissues**

**Supplementary Fig. 5 mRNA levels of inflammatory mediators in HaCaT cell line after EHMT2 knockdown.** (One-way ANOVA)

**Supplementary Fig. 6 mRNA levels of inflammatory mediators in NHEK after EHMT2 knockdown.** (One-way ANOVA)

**Graphical abstract A proposed working model on G9A function in keratinocyte.**

**Supplementary Table 1** **Related Sequences Table.**
