# Supplementary figures and images for: Depletion of G9A attenuates imiquimod-induced psoriatic dermatitis via targeting EDAR-NF-κB signaling in keratinocyte
Source: Cell Death Dis. 2023 Sep 22;14(9):627. doi: 10.1038/s41419-023-06134-y (PMC10517171; doi:10.1038/s41419-023-06134-y)

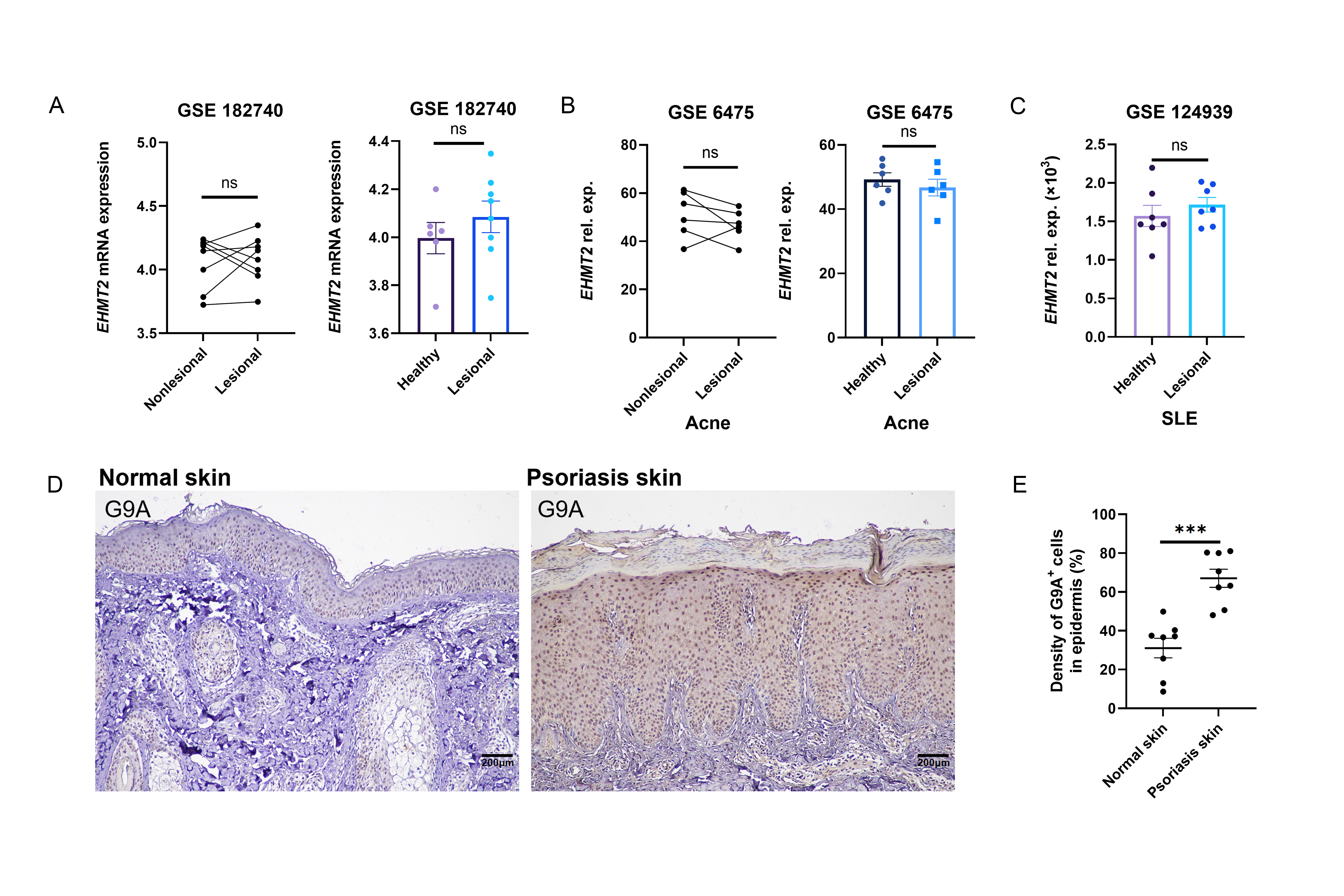

Supplement: Supplementary file 2 — Figure S1 [file 41419_2023_6134_MOESM2_ESM.png]

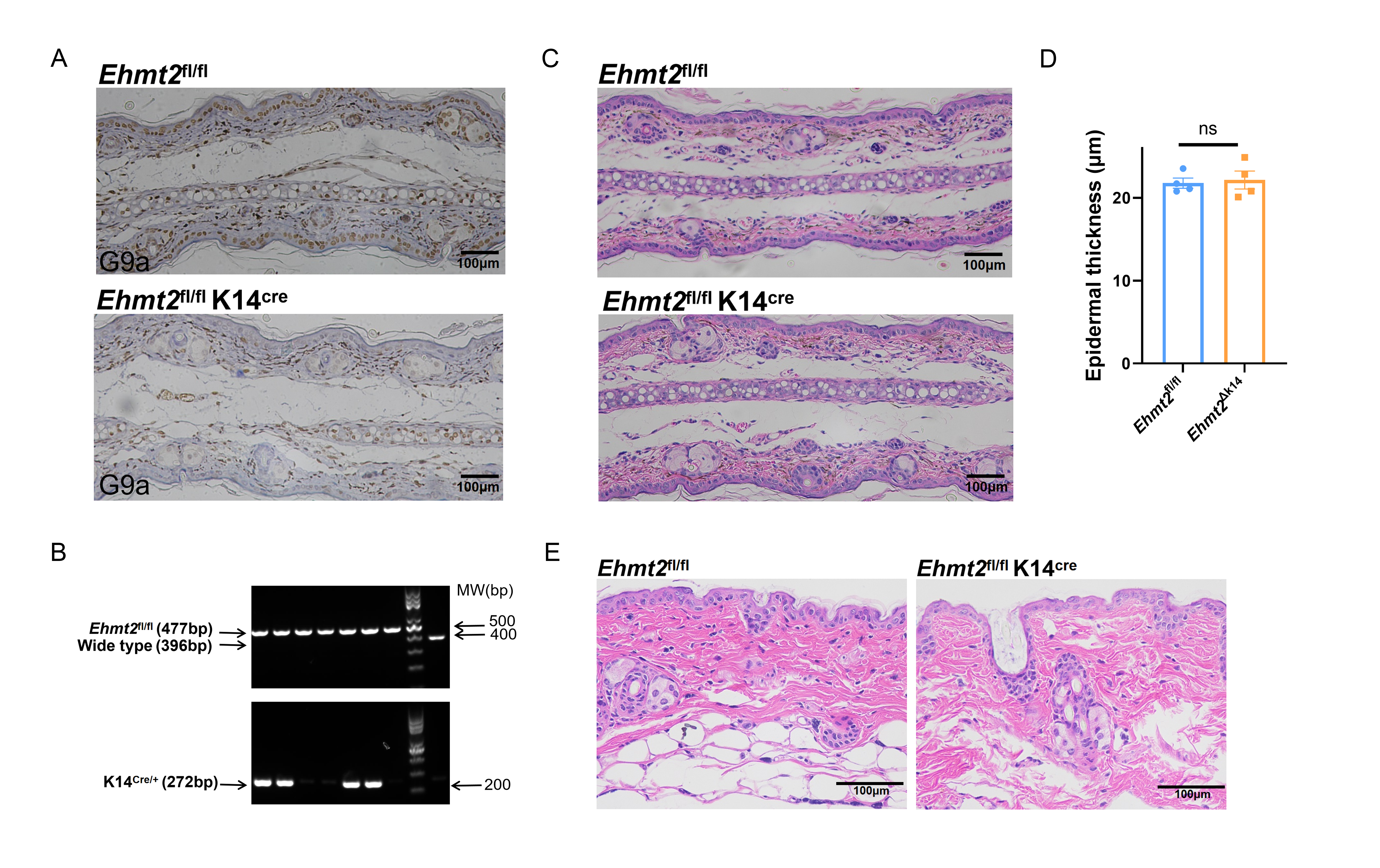

Supplement: Supplementary file 3 — Figure S2 [file 41419_2023_6134_MOESM3_ESM.png]

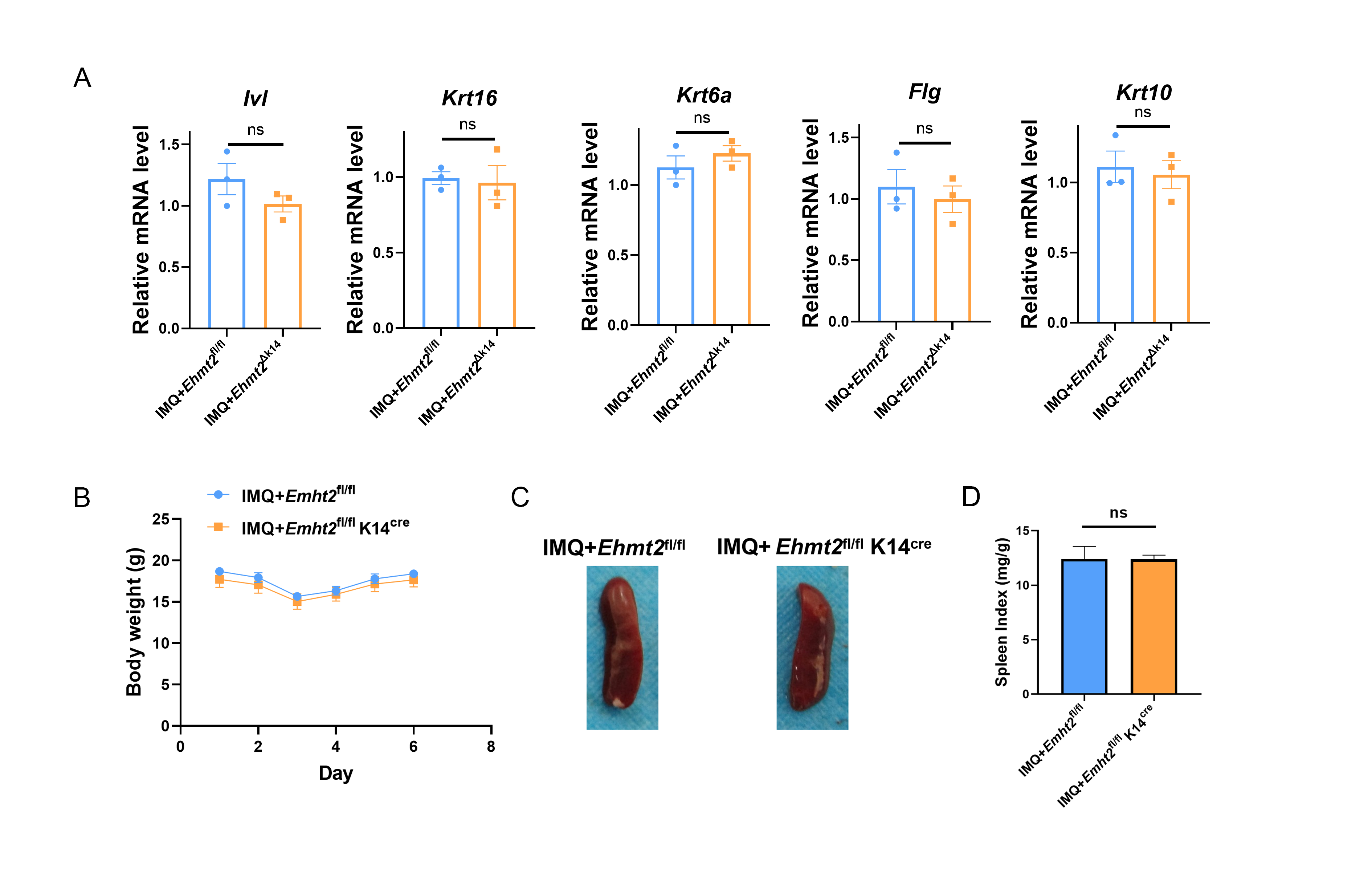

Supplement: Supplementary file 4 — Figure S3 [file 41419_2023_6134_MOESM4_ESM.png]

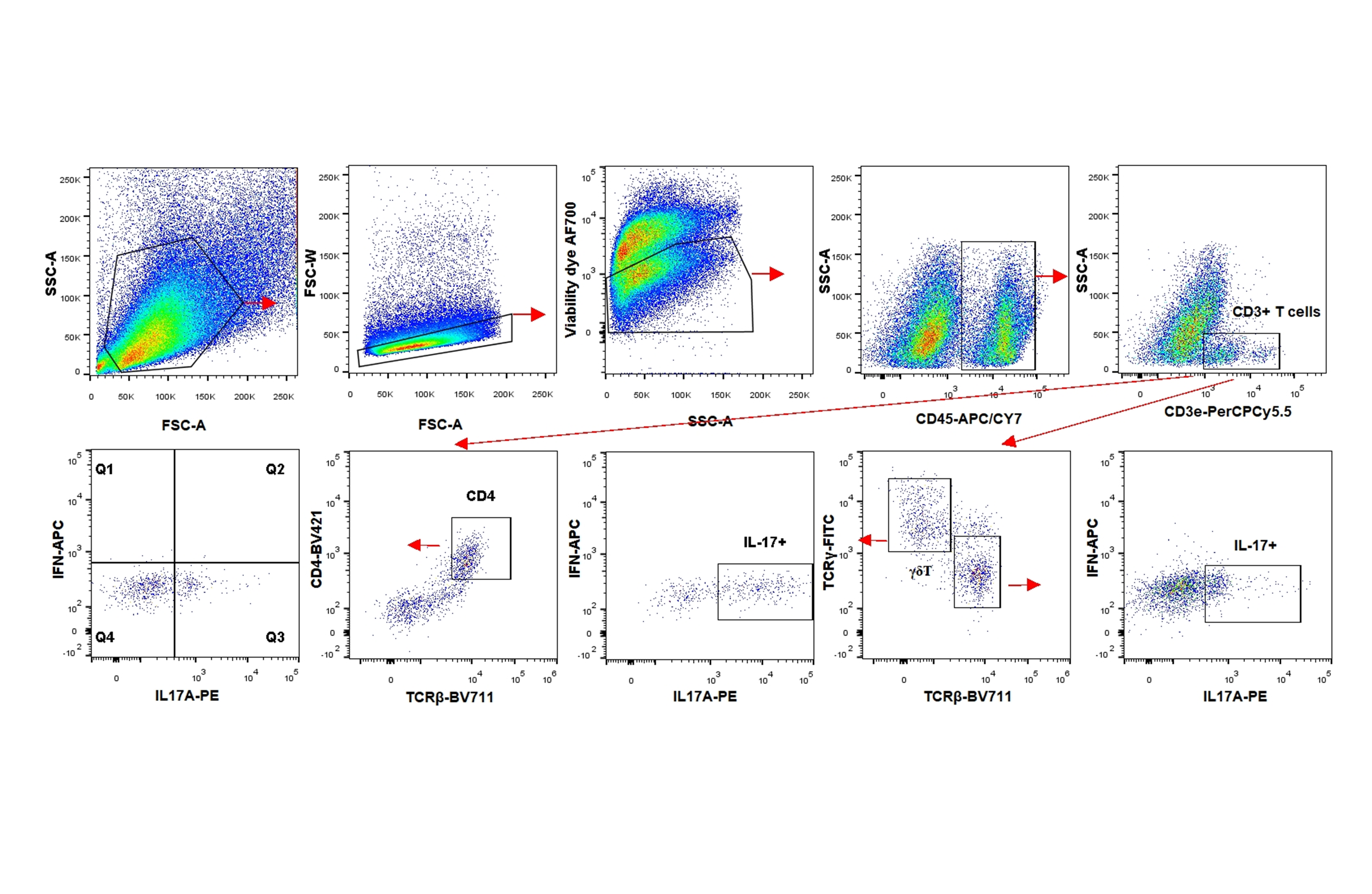

Supplement: Supplementary file 5 — Figure S4 [file 41419_2023_6134_MOESM5_ESM.png]

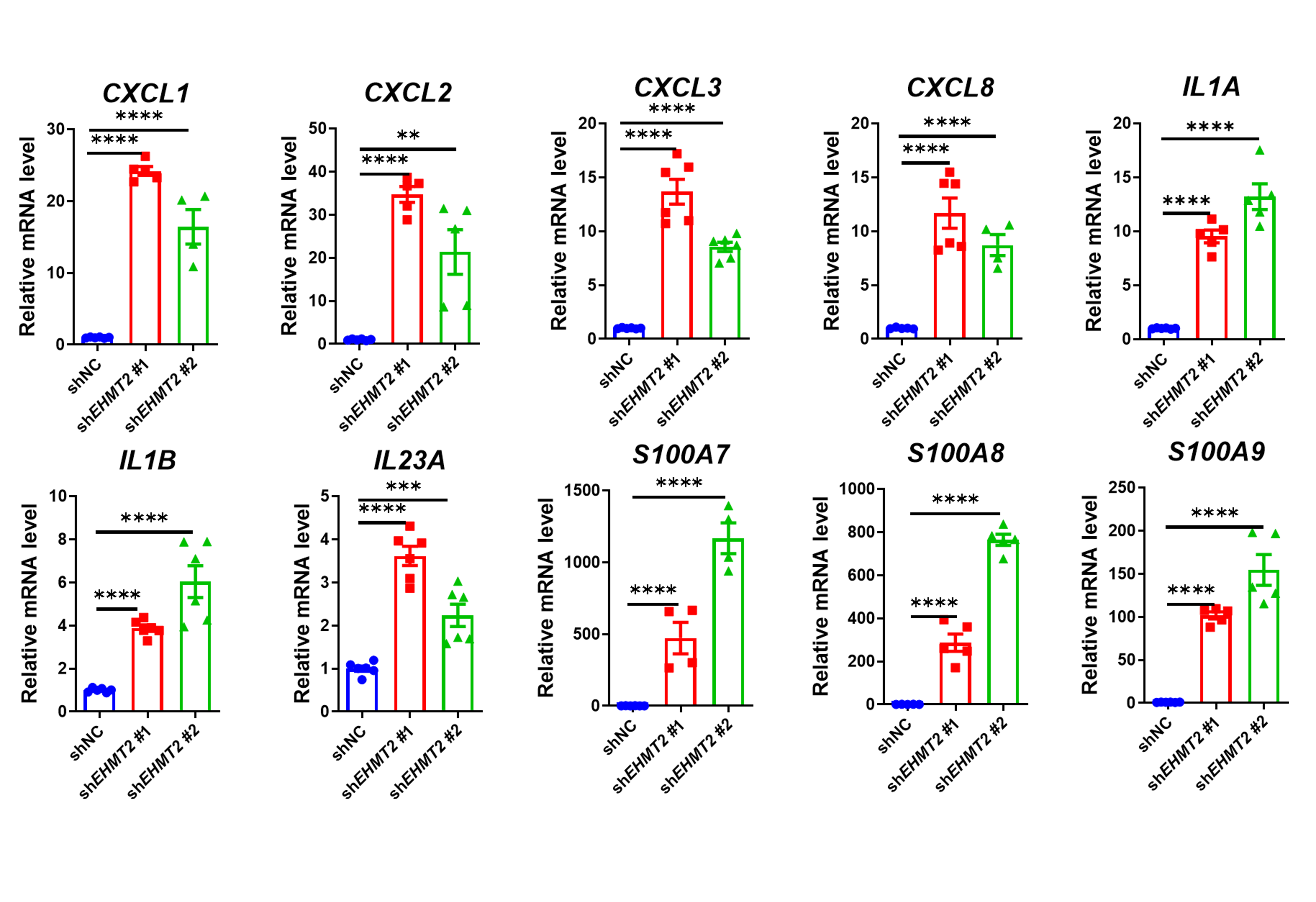

Supplement: Supplementary file 6 — Figure S5 [file 41419_2023_6134_MOESM6_ESM.png]

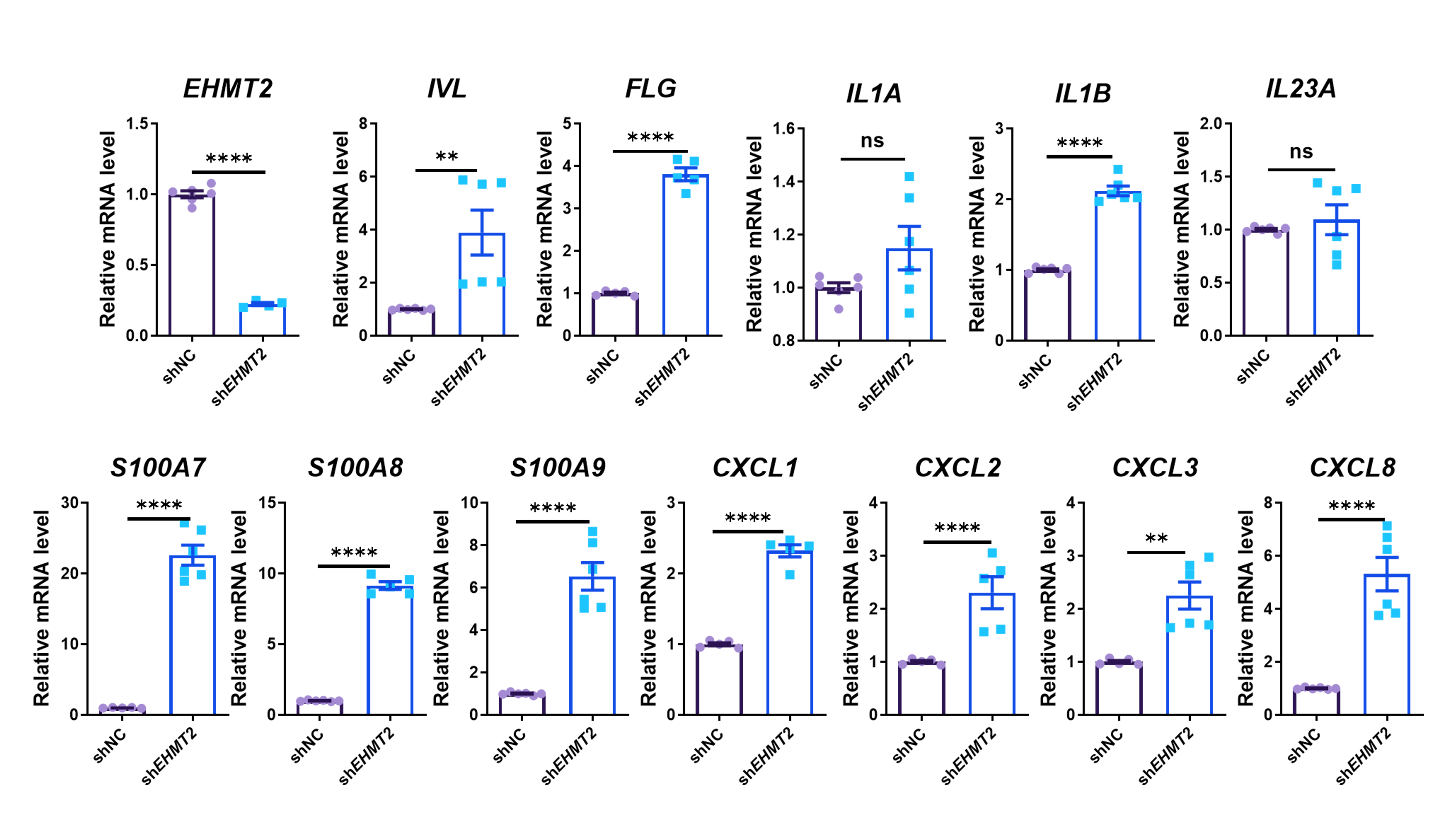

Supplement: Supplementary file 7 — Figure S6 [file 41419_2023_6134_MOESM7_ESM.png]
