# Supplementary material for: Depletion of G9A attenuates imiquimod-induced psoriatic dermatitis via targeting EDAR-NF-κB signaling in keratinocyte
Source: Cell Death Dis. 2023 Sep 22;14(9):627. doi: 10.1038/s41419-023-06134-y (PMC10517171; doi:10.1038/s41419-023-06134-y)
Supplement: Supplementary file 8 — Supplementary Table 1 [file 41419_2023_6134_MOESM8_ESM.docx]

**Supplementary Table 1** **Related Sequences Table**

| **q-PCR** | **Forward primer** | **Reverse primer** |
| --- | --- | --- |
| Mouse Gapdh | AGGTCGGTGTGAACGGATTTG | TGTAGACCATGTAGTTGAGGTCA |
| Mouse Ehmt2 | CCAGGAGTCTGAGAGGCGGAAG | CTGCTGGTCGCTCTGGAAGTTG |
| Mouse Il1b | ACGGACCCCAAAAGATGAAG | TTCTCCACAGCCACAATGAG |
| Mouse Il17f | TGCTACTGTTGATGTTGGGAC | AATGCCCTGGTTTTGGTTGAA |
| Mouse Il22 | AGCTTGAGGTGTCCAACTTC | GGTAGCACTGATCCTTAGCACTG |
| Mouse S100a8 | AGTGTCCTCAGTTTGTGCAG | ACTCCTTGTGGCTGTCTTTG |
| Mouse S100a9 | ATACTCTAGGAAGGAAGGACACC | TCCATGATGTCATTTATGAGGGC |
| Mouse Edar | CCAACTGTGGTGAGAACGAAT | TCGTCGTCTTTAGTGCCGTAT |
| Mouse Edaradd | GTCAGCAGAAACCAGCCCTGTAAG | AGCAAGTCCTGATCGTTGAGCAAG |
| Human GAPDH | GAAGGTGAAGGTCGGAGTCAA | GGAAGATGGTGATGGGATTTC |
| Human EHMT2 | GCCATCCGCACAGAGAAGATCATC | CCATCCACACCGTTGACACAGG |
| Human EDAR | CCATCGCCATCGTCCTCATCATC | CTTCTCCTCGTCCTTGCTCACTTG |
| Human EDARADD | AACTGTACTTGTTCCTCCTGCTTGC | GGGTGACACGGATCCAGCTTTATC |
| Human CXCL1 | TGCTGCTCCTGCTCCTGGTAG | GGGGACTTCACGTTCACACTTTGG |
| Human CXCL2 | TGCTGCTCCTGCTCCTGGTG | GGGGACTTCACCTTCACACTTTGG |
| Human CXCL3 | GCGTCCGTGGTCACTGAACTG | GCGTCCGTGGTCACTGAACTG |
| Human CXCL8 | GGACCACACTGCGCCAACAC | CCCTCTGCACCCAGTTTTCCTTG |
| Human IL1A | GACCAACCAGTGCTGCTGAAGG | GCCGTGAGTTTCCCAGAAGAAGAG |
| Human IL1B | CTCCACCTCCAGGGACAGGATATG | TCATCTTTCAACACGCAGGACAGG |
| Human IL23A | CTGAGGGTCACCACTGGGAGAC | TGGAGGCTGCGAAGGATTTTGAAG |
| Human S100A7 | CCCAACTTCCTTAGTGCCTGTGAC | GCTCTGCTTGTGGTAGTCTGTGG |
| Human S100A8 | TTGCTAGAGACCGAGTGTCCTCAG | GCCACGCCCATCTTTATCACCAG |
| Human S100A9 | GAACACATCATGGAGGACCTGGAC | GGTTAGCCTCGCCATCAGCATG |
| Human IVL | ACAAGGGAAGAGAGAGCCACTG | TGTAGAGGGACAGAGTCAAGTTCA |
| Human FLG | TGAAGCCTATGACACCACTGA | TCCCCTACGCTTTCTTGTCCT |

| **Genotyping** | **Forward primer** | **Reverse primer** |
| --- | --- | --- |
| Ehmt2-flox | TCAACCCTACTCACTTCAGTCAGC | CAAACAGAACACCCAAGGACA |
| K14-cre | AGCGATGGATTTCCGTCTCTGG | AGCTTGCATGATCTCCGGTATTGAA |

| **siRNA** | **Target sequence** |
| --- | --- |
| siRELA | AACAGTGTGTCATCCTTCT |
| SiNFKB1 | TAGTCTACATTTGAGACCG |

| **shRNA** | **Target sequence** |
| --- | --- |
| shEHMT2#1 | TCTTGAGCTTCAGAAGCATCC |
| shEHMT2#2 | GGTTGAGTTGAAGCGCAAACC |
