# Supplementary material for: Depletion of G9A attenuates imiquimod-induced psoriatic dermatitis via targeting EDAR-NF-κB signaling in keratinocyte
Source: Cell Death Dis. 2023 Sep 22;14(9):627. doi: 10.1038/s41419-023-06134-y (PMC10517171; doi:10.1038/s41419-023-06134-y)
Supplement: Supplementary file 11 — Authorship Change Approval [file 41419_2023_6134_MOESM11_ESM.pdf]

Authorship Change Approval

1. Mingzhu Yin

yinmingzhu2008@126.com (3136) | 设置 手机App 下载桌面端 参与调研 自助查询 续费

Cell Death & Disease

发件人：mingzhu<yinmingzhu2008@126.com>

收件人：Ming-Zhu Yin<yinmingzhu2008@126.com>

时　间：2023年09月07日 14:13 (星期四)

翻译成中文

Dear Dr. Mingzhu,

Since Dr. Bo Huang funded and directed the revision of this study submitted to *Cell Death & Disease*, we added his name as a co-corresponding author in the revised manuscript entitled "**Depletion of G9A attenuates imiquimod-induced psoriatic dermatitis via targeting EDAR-NF-κB signaling in keratinocyte**" (ID: CDDIS-23-2127RR). We would like to confirm with you whether you know the author list change.

Best regards!

Sincerely,

Mingzhu Yin, Ph.D., MD.

Professor and Chair

Clinical Research Center (CRC), Medical Pathology Center (MPC), Cancer Early Detection and Treatment Center (CEDTC), Chongqing University Three Gorges Hospital; Translational Medicine Research Center (TMRC), School of Medicine Chongqing University, Chongqing University, Chongqing, China.

yinmingzhu2008@126.com (3136) | 设置 手机App 下载桌面端 参与调研 自助查询 续费

Reply:Cell Death & Disease

发件人：mingzhu<yinmingzhu2008@126.com>

收件人：Ming-Zhu Yin<yinmingzhu2008@126.com>

时　间：2023年09月07日 14:15 (星期四)

翻译成中文

Dear Dr. Mingzhu,

Yes, I am aware of this and agree to this author list change.

Sincerely,

Mingzhu Yin, Ph.D., MD.

At 2023-09-07 14:13:16, "mingzhu" <yinmingzhu2008@126.com> wrote:

- 显示引用文字 -

--

Professor and Chair

Clinical Research Center (CRC), Medical Pathology Center (MPC), Cancer Early Detection and Treatment Center (CEDTC), Chongqing University Three Gorges Hospital; Translational Medicine Research Center (TMRC), School of Medicine Chongqing University, Chongqing University, Chongqing, China.

2. Zhiqin Fang

yinmingzhu2008@126.com (3137) | 设置 手机App 下载桌面端 参与调研 自助查询 续费

录 应用中心 收件箱 Cell De... Re: Cell D... Cell Death... PDF转换工具 Re: Cell D...

返回 回复 回复全部 转发 删除 举报 拒收 标记为 移动到 更多

Re: Cell Death & Disease 安全浏览模式 优化阅读 精简信息

发件人: 房智琴<208102091@csu.edu.cn>

收件人: mingzhu<yinmingzhu2008@126.com>

时 间: 2023年09月07日 14:17 (星期四)

翻译成中文

Dear Dr. Mingzhu,

Yes, I am aware of this and agree to this author list change.

Sincerely,

Zhiqin Fang

-----原始邮件-----

发件人: mingzhu <yinmingzhu2008@126.com>

发送时间: 2023-09-07 15:11:02 (星期四)

收件人: 208102091@csu.edu.cn

抄送:

主题: Cell Death & Disease

Dear Dr. Zhiqin,

Since Dr. Bo Huang funded and directed the revision of this study submitted to *Cell Death & Disease*, we added his name as a co-corresponding author in the revised manuscript entitled "**Depletion of G9A attenuates imiquimod-induced psoriatic dermatitis via targeting EDAR-NF-κB signaling in keratinocyte**" (ID: CDDIS-23-2127RR). We would like to confirm with you whether you know the author list change.

Best regards!

Sincerely,

Mingzhu Yin, Ph.D., MD.

Professor and Chair

Clinical Research Center (CRC), Medical Pathology Center (MPC), Cancer Early Detection and Treatment Center (CEDTC), Chongqing University Three Gorges Hospital; Translational Medicine Research Center (TMRC), School of Medicine Chongqing University, Chongqing University, Chongqing, China.

3. Yutong Wang

yinmingzhu2008@126.com (3131) | 设置 手机App 下载桌面端 参与调研 自助查询 续费 搜索邮件

通讯录 应用中心 收件箱 Re: Cell De... ×

<< 返回 回 复 回复全部 转 发 删 除 举 报 拒 收 标记为 移动到 更 多

Re: Cell Death & Disease 安全浏览模式 优化阅读 精简信息

发件人：8301200203@csu.edu.cn<8301200203@csu.edu.cn>

收件人：mingzhu<yinmingzhu2008@126.com>

时　间：2023年09月07日 14:27 (星期四)

翻译成中文

Dear Dr.Mingzhu,

Yes,I am aware of this and agree to this author list change.

Sincerely,

Yutong Wang.

发自我的iPhone

------------------ Original ------------------

From: mingzhu <yinmingzhu2008@126.com>
Date: Thu,Sep 7,2023 3:12 PM
To: 8301200203 <8301200203@csu.edu.cn>
Subject: Re: Cell Death & Disease

Dear Dr. Yutong,

Since Dr. Bo Huang funded and directed the revision of this study submitted to *Cell Death & Disease*, we added his name as a co-corresponding author in the revised manuscript entitled "**Depletion of G9A attenuates imiquimod-induced psoriatic dermatitis via targeting EDAR-NF-κB signaling in keratinocyte**" (ID: CDDIS-23-2127RR). We would like to confirm with you whether you know the author list change.

Best regards!

Sincerely,

Mingzhu Yin, Ph.D., MD.

Professor and Chair
Clinical Research Center (CRC), Medical Pathology Center (MPC), Cancer Early Detection and Treatment Center (CEDTC), Chongqing University Three Gorges Hospital; Translational Medicine Research Center (TMRC), School of Medicine Chongqing University, Chongqing University, Chongqing, China.

4. Bo Huang

yinmingzhu2008@126.com (3131) | 设置 手机App 下载桌面端 参与调研 自助查询 续费

应用中心 已发送 Re:Cell Dea... Cell Death ...

返回 再次编辑发送 回复全部 转发 删除 移动到 更多 撤回邮件

Cell Death & Disease 安全浏览模式 优化阅读 | 精简信息

发件人：mingzhu<yinmingzhu2008@126.com>

收件人：huangb2017<huangb2017@163.com>

时 间：2023年09月07日 14:09 (星期四)

发送状态：发送成功 查看详情

翻译成中文

Dear Dr. Bo Huang,

Since you funded and directed the revision of this study submitted to *Cell Death & Disease*, we added your name as a co-corresponding author in the revised manuscript entitled "**Depletion of G9A attenuates imiquimod-induced psoriatic dermatitis via targeting EDAR-NF-κB signaling in keratinocyte**" (ID: CDDIS-23-2127RR). We would like to confirm with you whether you know the author list change.

Best regards!

Sincerely,

Mingzhu Yin, Ph.D., MD.

Professor and Chair

Clinical Research Center (CRC), Medical Pathology Center (MPC), Cancer Early Detection and Treatment Center (CEDTC), Chongqing University Three Gorges Hospital; Translational Medicine Research Center (TMRC), School of Medicine Chongqing University, Chongqing University, Chongqing, China.

yinmingzhu2008@126.com (3131) | 设置 手机App 下载桌面端 参与调研 自助查询 续费

应用中心 收件箱 Re:Cell De... Cell Death ...

返回 回复 回复全部 转发 删除 举报 拒收 标记为 移动到 更多

Re:Cell Death & Disease 安全浏览模式 优化阅读 | 精简信息

发件人：Bo HUANG<huangb2017@163.com>

收件人：mingzhu<yinmingzhu2008@126.com>

时 间：2023年09月07日 14:25 (星期四)

Yes, I am aware of this and agree to this author list change.

Bo Huang

At 2023-09-07 15:09:09, "mingzhu" <yinmingzhu2008@126.com> wrote:

- 显示引用文字 -

5. Xiang Chen

yinmingzhu2008@126.com (3136) | 设置 手机App 下载桌面端 参与调研 自助查询 续费

录 应用中心 收件箱 Cell Dea... Re: Cell De... Cell Death ... PDF转换工具

返回 回复 回复全部 转发 删除 举报 拒收 标记为 移动到 更多

Re: Cell Death & Disease 安全浏览模式 精简信息

发件人：陈翔<chenxiangck@126.com>

收件人：印明柱<yinmingzhu2008@126.com>

时 间：2023年09月07日 14:09 (星期四)

翻译成中文

Dear Dr. Mingzhu,

Yes, I am aware of this and agree to this author list change.

Sincerely,

Xiang Chen, MD, PhD
Professor of Dermatology, Xiangya Hospital
Vice President of Central South University
President of Xiangya Medical College
Tel: +86 731-88879282
Fax: +86 731-88710591

---- Replied Message ----

From	mingzhu<yinmingzhu2008@126.com>
Date	9/7/2023 15:07
To	<chenxiangck@126.com>
Subject	Cell Death & Disease

Dear Dr. Xiang Chen,

Since Dr. Bo Huang funded and directed the revision of this study submitted to *Cell Death & Disease*, we added his name as a co-corresponding author in the revised manuscript entitled "**Depletion of G9A attenuates imiquimod-induced psoriatic dermatitis via targeting EDAR-NF-κB signaling in keratinocyte**" (ID: CDDIS-23-2127RR). We would like to confirm with you whether you know the author list change.

Best regards!

Sincerely,

Mingzhu Yin, Ph.D., MD

Professor and Chair
Clinical Research Center (CRC), Medical Pathology Center (MPC), Cancer Early Detection and Treatment Center (CEDTC), Chongqing University Three Gorges Hospital; Translat[illegible]al Medicine Research Center (TMRC), School of Medicine Chongqing University, Chongqing University, Chongqing, China.

6. Rundong Jiang

yinmingzhu2008@126.com (3131) | 设置 手机App 下载桌面端 参与调研 自助查询 续费

讯录 应用中心 收件箱 Re: Cell De...

返回 回复 回复全部 转发 删除 举报 拒收 标记为 移动到 更多

Re: Cell Death & Disease 安全浏览模式 优化阅读 精简信息

发件人: 姜润东<2204160417@csu.edu.cn>

收件人: mingzhu<yinmingzhu2008@126.com>

时 间: 2023年09月07日 14:28 (星期四)

翻译成中文

Dear Dr. Mingzhu,

Yes, I am aware of this and agree to this author list change.

Sincerely,

Rundong Jiang

Dermatology, Xiangya Hospital

——原始邮件——

发件人: mingzhu <yinmingzhu2008@126.com>

发送时间: 2023-09-07 15:10:02 (星期四)

收件人: 2204160417@csu.edu.cn

抄送:

主题: Cell Death & Disease

Dear Dr. Rundong,

Since Dr. Bo Huang funded and directed the revision of this study submitted to *Cell Death & Disease*, we added his name as a co-corresponding author in the revised manuscript entitled "**Depletion of G9A attenuates imiquimod-induced psoriatic dermatitis via targeting EDAR-NF-κB signaling in keratinocyte**" (ID: CDDIS-23-2127RR). We would like to confirm with you whether you know the author list change.

Best regards!

Sincerely,

Mingzhu Yin, Ph.D., MD.

Professor and Chair

Clinical Research Center (CRC), Medical Pathology Center (MPC), Cancer Early Detection and Treatment Center (CEDTC), Chongqing University Three Gorges Hospital; Translational Medicine Research Center (TMRC), School of Medicine Chongqing University, Chongqing University, Chongqing, China.
