# Supplementary material for: Depletion of G9A attenuates imiquimod-induced psoriatic dermatitis via targeting EDAR-NF-κB signaling in keratinocyte
Source: Cell Death Dis. 2023 Sep 22;14(9):627. doi: 10.1038/s41419-023-06134-y (PMC10517171; doi:10.1038/s41419-023-06134-y)
Supplement: Supplementary file 10 — Original Data File [file 41419_2023_6134_MOESM10_ESM.docx]

**Original membranes**

**Figure 5H**

**Bcl-2**

**Bax**

**Caspase3**

**Cleaved-caspase3**

**G9a**

**Gapdh**

The uncropped western blot results in Figure 5H.

**Figure 6E**

**NFKB1**

**P65**

**Pho-p65**

**Traf6**

**Edar**

**Edaradd**

**Gapdh**

The uncropped western blot results in Figure 6.

**Figure 7H**

**Bcl2**

**Nfkb1**

**Pho-p65**

**Caspase3**

**Actin**

**Figure 7I**

**Edar**

**Caspase3**

**Bcl-2**

**Bax**

**Tubulin**

**Pho-p65**

**NFKB1**

The uncropped western blot results in Figure 7I.
